# Reshaping the tumor microenvironment: The versatility of immunomodulatory drugs in B-cell neoplasms

**DOI:** 10.3389/fimmu.2022.1017990

**Published:** 2022-10-12

**Authors:** Hao Guo, Jingyi Yang, Haoran Wang, Xingchen Liu, Yanyan Liu, Keshu Zhou

**Affiliations:** Department of Hematology, The Affiliated Cancer Hospital of Zhengzhou University & Henan Cancer Hospital, Zhengzhou, China

**Keywords:** Immunomodulatory drug, B-cell lymphoma, Multiple myeloma, Tumor microenvironment, Immunotherapy, CRBN

## Abstract

Immunomodulatory drugs (IMiDs) such as thalidomide, lenalidomide and pomalidomide are antitumor compounds that have direct tumoricidal activity and indirect effects mediated by multiple types of immune cells in the tumor microenvironment (TME). IMiDs have shown remarkable therapeutic efficacy in a set of B-cell neoplasms including multiple myeloma, B-cell lymphomas and chronic lymphocytic leukemia. More recently, the advent of immunotherapy has revolutionized the treatment of these B-cell neoplasms. However, the success of immunotherapy is restrained by immunosuppressive signals and dysfunctional immune cells in the TME. Due to the pleiotropic immunobiological properties, IMiDs have shown to generate synergetic effects in preclinical models when combined with monoclonal antibodies, immune checkpoint inhibitors or CAR-T cell therapy, some of which were successfully translated to the clinic and lead to improved responses for both first-line and relapsed/refractory settings. Mechanistically, despite cereblon (CRBN), an E3 ubiquitin ligase, is considered as considered as the major molecular target responsible for the antineoplastic activities of IMiDs, the exact mechanisms of action for IMiDs-based TME re-education remain largely unknown. This review presents an overview of IMiDs in regulation of immune cell function and their utilization in potentiating efficacy of immunotherapies across multiple types of B-cell neoplasms.

## 1 Introduction

B-cell neoplasms, which stem from distinct stages of B-cell development, are a heterogeneous set of cancers including B-cell lymphomas (BCLs), chronic lymphocytic leukemia (CLL), and plasma cell dyscrasias such as multiple myeloma (MM) (1). Despite great advances have been achieved in diagnosis and treatment, these hematologic disorders still cause significant global morbidity and mortality. The introduction of a safe and more effective new class of drugs, especially the monoclonal antibodies (mAbs) (e.g. anti-CD20 rituximab and anti-CD38 daratumumab), has made remarkable therapeutic progress in the past twenty years. Yet a large number of patients still fail to have response or relapse eventually. More recently, novel immunotherapies including immune checkpoint inhibitors (ICIs) and chimeric antigen receptor (CAR) T-cell therapy have made breakthroughs in treatment of refractory disease (2, 3). However, the success of immunotherapy is impeded by inhibitory signals which reside in cancer cells or that are generated from the tumor microenvironment (TME), which restricts the tumor-suppressive capacity of the immune system (4–6).

TME is a complex network consisting of both cellular and non-cellular compositions, which forms a physical barrier around malignant cells. Increasing evidence has established that components of TME play vital roles in a series of processes of tumor development, including carcinogenesis, progression, metastasis and treatment resistance (6–8). Recognition of the TME has paved the way for exploring novel strategies targeting the microenvironment as well as its interplays with tumor cells (9). Immunomodulatory drugs (IMiDs) are a group of anticancer agents including thalidomide and its analogs lenalidomide and pomalidomide. These compounds show pleiotropic effects in hematologic malignancies including anti-angiogenic, anti-proliferative and immunobiologic properties by direct cytotoxicity towards tumor cells and indirectly interfering with cellular components of the TME (10–12). Herein, we provide a comprehensive review of the immunomodulatory activities of thalidomide analogues towards T cells, tumor-associated macrophages (TAMs), natural killer (NK) cells, dendritic cells (DCs) and stromal cells. In addition, we also discuss the clinical efficacy of IMiDs in combination with the state-of-the-art immunotherapies to shed light on optimal TME–targeted treatment strategy.

## 2 Development of IMiDs

### 2.1 Drug repurposing and regeneration

**Figure 1:**
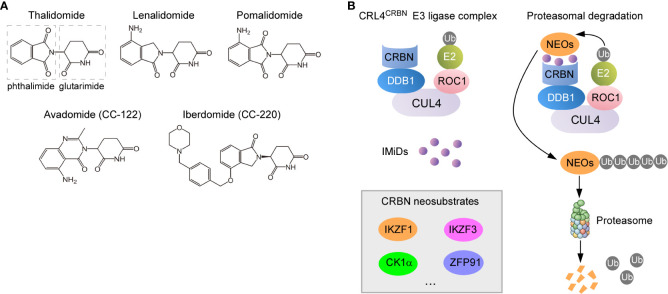
Molecular mechanisms of action for IMiDs. **(A)** Structure of thalidomide and its analogues. They all share a glutarimide ring that binds to CRBN while vary in the neosubstrate-binding moiety (phthaloyl ring). **(B)** Proteasomal degradation of CRBN neosubstrates redirected by IMiDs. IMiDs act as the molecular glue to recruit neosubstrate proteins to CRBN receptor component of the CRL4^CRBN^ E3 ligase complex (left), which leads to the sequential ubiquitylation and degradation of neosubstrates (NEOs) (right).

Thalidomide (α-N-phthalimido-glutarimide) (**Figure 1A**), a synthetic glutamic acid derivative, was once infamous for its potent teratogen causing dysmelia when used for alleviating nausea during pregnancy in the late 1950s and early 1960s. Despite withdrawal from markets that time, thalidomide regained its new life four decades later when immunomodulatory and anti-tumor effects were discovered (10, 13, 14). The first evidence for the immunomodulatory functions of thalidomide was demonstrated that it was effective in the treatment of erythema nodosum leprosum due to its ability to inhibit TNFα secreted by activated monocytes (15, 16). Except for this anti-inflammatory property, thalidomide was subsequently shown to exert other immunomodulatory properties such as co-stimulation of T cells and activation of NK cells (17). Along with these findings, the recognition of thalidomide as an inhibitor of angiogenesis further fueled a surge of interest in repurposing thalidomide as a promising anti-neoplastic therapy (18). As such, a set of formal medicinal chemistry programs were then initiated to discover novel derivatives with enhanced efficacy while less toxicity compared with thalidomide (19). Lenalidomide and pomalidomide (**Figure 1A**), the two first-in-class IMiDs, are derived by adding an amino group to the fourth carbon of the phthaloyl ring of thalidomide (13).

Lenalidomide was the first thalidomide analogue developed, consequently dominating the clinical development in hematologic malignancies (14). Lenalidomide was also the first agent of this group of immunomodulatory drugs approved by US Food and Drug Administration (FDA) for the treatment of MM, relapsed/refractory (R/R) mantle cell lymphoma (MCL), and myelodysplastic syndrome (MDS) with deletion 5q (20–24). Recently, it has been approved for previously treated follicular lymphoma (FL) and marginal zone lymphoma (MZL) in combination with rituximab (25–27). Notably, in 2020, lenalidomide combined with tafasitamab (a CD19 targeting mAb) received accelerated approval for patients with R/R diffuse large B-cell lymphoma (DLBCL) (28).

As the third-generation thalidomide analogue, pomalidomide contains both the phthalimide and the glutarimide moieties like thalidomide but differs in an amino substituent at the four position of the phthalimide ring (**Figure 1A**) (29). Pomalidomide has been approved for the treatment of MM, which is more powerful than lenalidomide and shows efficacy in cases that are resistant to lenalidomide (30, 31). Furthermore, it is now under extensive exploration in preclinical or clinical studies on aggressive BCLs including DLBCL, primary effusion lymphoma (PEL) and primary central nervous system lymphoma (PCNSL) (32–37). Avadomide (also called CC-122) (**Figure 1A**), a novel modulator of cereblon E3 ubiquitin ligase (CELMoD) exhibiting potent anti-lymphoma and immunomodulatory activities, is currently in phase I trials (38, 39). Other new CELMoDs such as CC-220 (iberdomide) and CC-885 (**Figure 1A**) have shown efficacy in the treatment of systemic lupus erythematosus (SLE) and acute myeloid leukemia (AML) (40–42). The established applications and most common side effects of three approved IMiDs (thalidomide, lenalidomide and pomalidomide) are summarized in **Table 1**.

**Table 1:** Applications of thalidomide analogues in hematologic malignancies and reported toxicities.

	Thalidomide	Lenalidomide	Pomalidomide
**Preclinical activities**	MM (43–45) NHL (46) CLL (47, 48) AML (49–53) ALL (54, 55)	MM (56–61) NHL (11, 61–71) CLL (72–77) AML (78, 79) MDS (78, 80–83)	MM (61, 84–87) NHL (34, 35, 37, 61, 88) AML (40, 89)
**Clinical applications**	MM* (90–95) FL (96, 97) MCL (98–100) HL (101, 102) TCL (103, 104) CLL (105–108) DLBCL (109) MALT lymphoma (110) AML (111–113) MDS (111, 114–117) CMML (118) CML (119)	MM* (120–125) MDS* (126, 127) MCL* (128–132) FL* (25–27, 128, 133–135) MZL* (26, 27, 128, 135) SLL (26, 27, 128, 135) CLL (136–141) DLBCL (128, 142, 143) MALT lymphoma (110, 144) PCNSL (145, 146) TCL (147–150) AML (127, 151–155) CMML (156–159)	MM* (160–163) CLL (164) DLBCL (32, 164) PCNSL (33) MPN (165, 166) MDS (167) AML (40, 167, 168)
**Toxicities**	Teratogenicity (169) Constipation (169) Hypothyroidism (169) ACTH stimulation (169) Hypoglycemia (169) Xerostomia (169) Fever (169) Mood changes (169) Headache (169) Peripheral neuropathy (169) Somnolence (169) Sedation (169) Rash (169) VTE (169)	Neutropenia (121) Anemia (121) Thrombocytopenia (121) Diarrhea (121) Fatigue (121) Muscle cramps (121) Rash (121) Infections (121) VTE (121) Myelosuppressive effects (170) Secondary MDS/AML (171) Secondary ALL (172)	Neutropenia (170) Anemia (170) Thrombocytopenia (170) Fatigue (170) VTE (170) Neuropathy (170) Infections (170)

MM, Mutiple myeloma; NHL, Non-Hodgkin lymphoma; CLL, Chronic lymphocytic leukemia; AML, Acute myeloid leukemia; ALL, Acute lymphoblastic leukemia; MDS, Myelodysplastic syndrome; FL, Follicular lymphoma; MCL, Mantle cell lymphoma; HL, Hodgkin lymphoma; TCL, T-cell lymphoma; DLBCL, Diffuse large B-cell lymphoma: MALT lymphoma, Mucosa‐associated lymphoid tissue lymphoma; MZL, Marginal zone lymphoma; SLL, Small lymphocytic lymphoma; PCNSL, Primary central nervous system lymphoma; CMML, Chronic myelomonocytic leukemia; MPN, Myeloproliferative neoplasm; ACTH, Adrenocorticotropic hormone; VTE, Venous thromboembolism. *, FDA-approved applications.

### 2.2 Mechanism of action

IMiDs exert their anti-tumor effects by a unique mechanism of action (MOA), not only killing the malignant cells directly, but also modulating nonmalignant immune cells (T cells, NK cells, TAMs, DCs etc.) within the TME, which are believed to contribute to lymphoma progression and survival (10, 11, 13). Due to the pleiotropic effects of IMiDs, their molecular targets were believed to be various. The direct target of IMiDs was unknown until Ito et al. identified cereblon (CRBN) as the sole molecular target underlying thalidomide teratogenicity (173). Thereafter, various studies have focused on elucidating the role of CRBN in the effects of thalidomide analogues, especially for lenalidomide (56, 80, 174–176). As a result, CRBN is currently regarded as a primary direct target for therapeutic activities of all IMiDs (13).

CRBN forms a cullin-4 RING E3 ubiquitin ligase complex (CRL4^CRBN^) with DNA damage-binding protein 1 (DDB1), cullin 4 (CUL4), and regulator of cullins-1 (ROC1) (**Figure 1B**) (173, 177, 178). When bound by thalidomide derivatives, CRBN triggers protein ubiquitination and degradation of drug-specific neosubstrates. Substrate selectivity rests with the structure of IMiDs bond to CRBN (13, 179). IMiDs have a conserved glutarimide moiety that directly docks into a tri-tryptophan pocket on the surface of CRBN, which in turn activates its E3 ligase activity, modulates specificity of protein substrate and avoids autoubiquitylation (180, 181). In malignant B cells, IMiDs retarget CRBN-dependent ligase activity to Ikaros (IKZF1) and Aiolos (IKZF3), both of which are zinc finger–containing transcription factors in lymphoid development, resulting in their proteasomal degradation (14, 56, 88, 182, 183) (**Figure 1B**). The reduced abundance of Ikaros and Aiolos elicits direct anti-proliferative and anti-neoplastic effects against tumor cells. More importantly, a constellation of immunomodulatory effects arising from Ikaros and Aiolos degradation have been proposed to contribute to activities of IMiDs (14, 19), which include improved formation of immune synapse (IS) (184), potentiated co-stimulation of T cells (57), and enhanced release and function of anti-tumor cytokines (185).

It should be noted that different neosubstrate spectrum that are targeted for proteasomal degradation may account for the distinct activity of each thalidomide derivative (14). For instance, lenalidomide degrades casein kinase 1 alpha (CK1α, encoded by *CSNK1A1* gene) more efficiently than thalidomide and pomalidomide in myeloid neoplasms, thus providing a therapeutic window for lenalidomide in del (5q) MDS, where *CSNK1A1* haploinsufficiency due to genetic deletion sensitizes tumor cells to lenalidomide (80, 186, 187). A recent study showed that treatment with lenalidomide but not pomalidomide leads to expansion of pre-leukemic *Trp53*-mutant hematopoietic stem and progenitor cells (HSPCs) due to selective degradation of Ck1α, which offers a potential alternative strategy to mitigate the risk of therapy-related myeloid neoplasms (t-MNs) development (171). Accordingly, the efficacy and toxicity profiles of each IMiD and the precise use of these agents need to be thoroughly investigated.

## 3 The anti-tumor activities of IMiDs

### 3.1 Direct effects on malignant B cells

Direct anti-neoplastic activity of IMiDs against malignant B cells has been demonstrated in MM, CLL and aggressive non-Hodgkin lymphoma (NHLs) (12, 188). Degradation of Ikaros and Aiolos by lenalidomide and pomalidomide leads to specific and sequential downregulation of c-Myc followed by interferon regulatory factor 4 (IRF4), which results in subsequent cell death of myeloma cells (189). In addition, lenalidomide can upregulate p21WAF/Cip1 expression and lead to cell cycle arrest in CLL cells (72). In Namalwa CSN.70, a Burkitt’s lymphoma cell line with chromosome 5 deletion, lenalidomide was shown to induce cell cycle arrest and inhibit Akt and Gab1 phosphorylation (190). Moreover, lenalidomide kills activated B cell-like (ABC) DLBCL cells by inhibiting IRF4 and the Ets transcription factor Spi-B while stimulating IFNβ production in a CRBN-dependent manner (191).

### 3.2 Pleiotropic effects of IMiDs on TME

**Figure 2:**
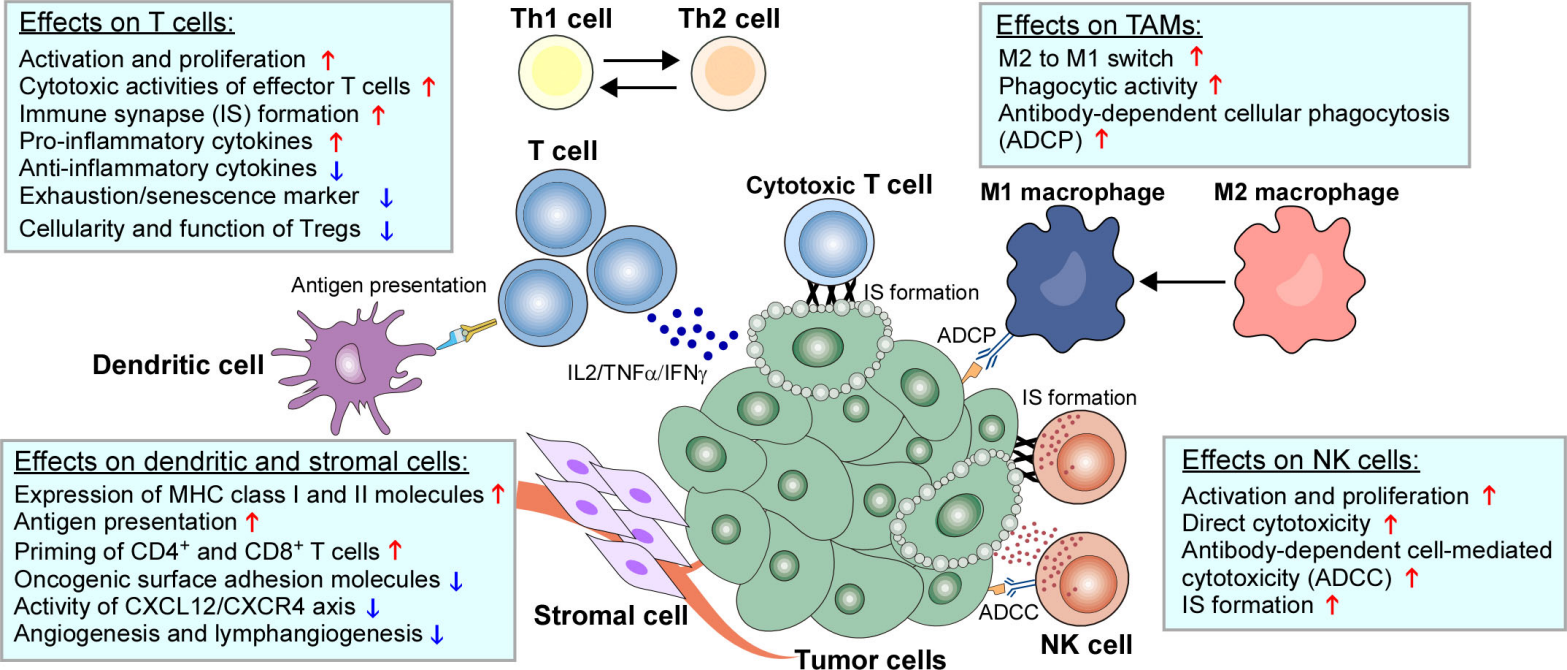
Immunomodulatory effects of IMiDs on the TME of B-cell neoplasms. The biological impacts of IMiDs on T cells, natural killer (NK) cells, tumor-associated macrophages (TAMs), dendritic cells and stromal cells are depicted.

Beyond the direct cytotoxicity towards malignant B cells, recent studies have emphasized the therapeutic implications of IMiDs-remodeled interplay between malignant cells and non-malignant immune cells in the TME within the lymph nodes and bone marrow (11, 12, 192). Despite these nursing cells usually build a supportive network for tumor development and drug resistance, they also have potential to drive antitumor immune responses in specific cases (5, 6). Early studies based on gene expression signature of FL patients found that the length of survival was associated with the molecular features of tumor-infiltrating immune cells at diagnosis, which was independent of clinically prognostic variables (193). This evidence was supported by direct studies demonstrating that TME cells such as follicular dendritic cells (FDCs), CD4^+^ T cells and bone marrow stromal cells promoted lymphoma cell survival and proliferation (194, 195). In addition, tumor-associated monocytes/macrophages can attract and work in concert with other immune cells (e.g. T cells) by secretion of chemokines CCL3 and CCL4 (196, 197). As a result, TME shields malignant B cells from the immune recognition and elimination. The underlying mechanisms include the dampened expression of molecules (e.g. MHC I and II) required for interactions with immune cells, defected T-cell IS formation, and the recruitment of immunosuppressive cells such as regulatory T cells (Tregs) and TAMs (198–200). The immunomodulatory effects of IMiDs on the TME, especially the immune cells, are summarized in **Table 2** and illustrated in **Figure 2**.

**Table 2:** Modulatory effects of IMiDs on immune cells and implications for improving immunotherapies.

Cell types	Effects of IMiDs	Rational combinations with immunotherapies
T cells	1. Promoting co-stimulation and proliferation (175, 201) 2. Enhancing T-cell effector functions (153, 202) 3. Increasing pro-inflammatory cytokine levels (192) 4. Improving IS formation between T cells and tumor cells (65) 5. Inhibiting T-cell exhaustion and senescence (192, 203) 6. Modulating Th1/Th2 subsets and Treg function (201, 204, 205)	1. Anti-PD-1/PD-L1 therapy (59, 206) 2. CAR-T cell therapies (73, 207, 208) 3. Bi-specific T-cell engager (209–211)
NK cells	1. Increasing NK-cell number (212) 2. Stimulating NK-cell activation (216–218) 3. Enhancing NK-cell cytotoxicity (217, 218, 221, 222) 4. Restoring IS formation (217) 5. Promoting ADCC (62)	1. Monoclonal antibodies (25, 26, 213–215) 2. Bispecific antibodies (219, 220)
TAMs	1. Switching M2 to M1 type (35, 223) 2. Enhancing phagocytosis (35) 3. Promoting ADCP (213, 225)	1. Monoclonal antibodies (25, 215, 224–227) 2. Bispecific antibodies (227, 228)
DCs	1. Promoting antigen uptake antigen and presentation (229) 2. Increasing expression of MHC class I and II molecules (229) 3. Enhancing T-cell priming by DCs (229) 4. Potentiating DC-mediated T-cell responses (229)	1. DC vaccination (230–233) 2. Anti-PD-1/PD-L1 therapy (234)

#### 3.2.1 Effects on T cells

Compelling evidence suggests that malignant B cells can induce an immune-suppressed, largely exhausted and senescent T-cell phenotype through numerous mechanisms, such as upregulation of inhibitory ligands, downregulation of co-stimulatory molecules and production of immunosuppressive cytokines, which ultimately results in suppression the T-cell surveillance and immune escape (199, 235–237).

Preclinical studies have shown that treatment with IMiDs enhances co-stimulation and proliferation of T cells by inducing pro-inflammatory cytokine (e.g. IFN-γ, TNF-α and IL-2), decreasing anti-inflammatory cytokines (e.g. IL-6 and IL-10) and potentiating DC-antigen presentation in MM and CLL (12, 192, 238, 239). The degradation of Ikaros and Aiolos by IMiDs relieves the transcriptional repression of *Il2* promoter, thus promoting IL-2 production (175). Moreover, IMiDs can reduce immune tolerance of myeloma cells by binding to B7 co-stimulation molecular and activating B7-CD28 pathway (240). IMiDs can also upregulate transcriptional activity of DNA-binding protein AP-1 to increase T-cell cytokine production (212, 240, 241). These mechanisms collectively contribute to a primed T-cell activation (212, 242).

Due to the influence of malignant B cells, tumor-infiltrating CD4^+^ and CD8^+^ T cells usually display decreased IS formation and effector function (11). Ex vivo lenalidomide treatment of T cells co-cultured with CLL or FL cells repairs IS formation defect by restoring T-cell actin cytoskeletal signaling and enhancing actin polymerization (184, 198, 202). In addition, lenalidomide was shown to induce actin reorganization and γδT-MCL IS formation, as well as expansion and cytotoxicity of γδT cells against MCL (11). Another study reported that lenalidomide can repair defected T-cell adhesion and migration in CLL by restoring normal levels of Rho-GTPase family (Rho, Rac1 and Cdc42) and rescuing LFA-1 function (243).

Clinical investigations also provided evidence for the positive regulation of IMiDs on T-cell functions. Lenalidomide maintenance therapy after autologous stem-cell transplantation (ASCT) increases CD8^+^ T-cell numbers, upregulates co-stimulatory molecules and reduce inhibitory checkpoint molecules in MM patients (244). Similarly, Danhof et al. showed that lenalidomide maintenance post ASCT preserves CD8^+^ T cells and reduces expression of PD-1, enabling synergetic efficacies with ICIs (203). These findings were further validated in patient-derived xenograft (PDX) models showing an enhanced anti-CLL activity by combining avadomide and anti-PD-1 or anti-PD-1 ligand (PD-L1) (245). Moreover, the tumor-promoting Th17/Th1 and Th22 cells and related cytokines (IL-17, IL-6, IL-1β etc.) were decreased in MM patients treated with IMiDs during induction chemotherapy compared to untreated patients, which was associated with a favorable clinical outcome (246). As a result, lenalidomide and obinutuzumab combination was shown to induce an activated T-cell phenotype and reshape gene signatures into effector memory T cell features in FL patients (202). While *in vitro* studies showed that lenalidomide and pomalidomide strongly inhibit generation, proliferation and function of Tregs possibly due to decreased FOXP3 expression, the impact of IMiDs on the cellularity of Tregs in patients with B-cell neoplasms remains controversial (11, 192). In a post-transplant MM setting, treatment with IMiDs during induction therapy pre-ASCT resulted in decreased Tregs while increased CD8^+^ T cells in peripheral blood (247). In contrast, another study showed that lenalidomide maintenance after ASCT increased Treg numbers in relapsed MM patients (204). A similar pattern was observed in MCL patients treated with lenalidomide (248).

#### 3.2.2 Effects on NK cells

NK cells are predominant innate lymphocytes that reject types of tumors and clear microbial infections (249), and more importantly, mediate antibody-dependent cell-mediated cytotoxicity (ADCC) against BCLs, which serves as the one of the major cytotoxic mechanisms for anti-CD20 mAb Rituximab (250). Numerous studies have demonstrated that the activity and function of NK cells can be potentiated by IMiDs in B-cell malignancies (212, 251). Lenalidomide treatment can increase NK-cell number, stimulate NK-cell activation, restore IS formation, and enhance direct NK-cell cytotoxicity as well as NK-dependent ADCC (212, 217, 221, 222, 234, 252). Mechanistically, the effect of lenalidomide on NK cells may be mediated indirectly *via* IL-2 produced by T cells. Either T-cell depletion or IL-2 blockade can completely abrogate NK-cell proliferation and cytotoxicity (212). The increased IL-2 and activation of NK cells correlate to increased IFN-γ synthesis and upregulation of CD69 (253). A recent study by Hideshima et al. demonstrated that pomalidomide directly binds to zeta-chain-associated protein kinase-70 (Zap-70) and triggers its phosphorylation to activate NK cells in a CRBN-independent manner. In addition, they also demonstrated a second mechanism whereby pomalidomide directly triggers granzyme-B and NK cytotoxicity which is mediated by CRBN-IKZF3 axis (218). Consistently, avadomide has shown to promote NK-cell proliferation and cytotoxicity by inducing IL-2 secretion and upregulating granzyme B and NKG2D receptor (254–256).

Lenalidomide was shown to enhance NK-dependent ADCC in BCL cell lines treated with rituximab (62). In this context, the increased expression of granzyme B and Fas ligand (FasL) may account for enhanced ADCC, which could be inhibited by a granzyme B inhibitor or FasL antibody (62). Moreover, lenalidomide lowers NK-cell activation thresholds by rituximab, thus augmenting NK-cell responses (217). On the other hand, lenalidomide synergistically enhances rituximab-induced phosphorylation of JNK and activates the mitochondrial apoptotic pathway in MCL cells (63). *In vivo* studies using immunodeficient mice inoculated with MCL cells demonstrated that lenalidomide and rituximab combination decreased tumor burden and prolonged animal survival along with the increased number of splenic NK cells (63). These data provide compelling proof-of-concept for the clinical translation of lenalidomide combination with rituximab into B-cell lymphoma treatment.

#### 3.2.3 Effects on TAMs

TAMs are the key cellular components of TME, which can produce chemokines, cytokines and growth factors to recruit immunosuppressive cells and support tumor progression (257–259). TAMs are typically classified into M1-like (anti-tumorigenesis) and M2-like (pro-tumorigenesis) types based on their different surface markers, gene expression signatures and metabolic traits. The conversion between M1 and M2 is a dynamic process named “macrophage polarization” which occurs in response to TME signals (257, 260). Repolarization of M2-like macrophages to M1 phenotype represents a novel promising therapeutic strategy (261).

A recent study showed that lenalidomide altered the M1/M2 polarization in myeloma-associated macrophages (MAMs) from MM patients. Mechanistically, lenalidomide interferes epigenetically with IRF4 and IRF5 *via* degradation of IKZF1 and shifts M2-like MAMs to a pro-inflammatory and tumoricidal phenotype that resemble M1 cells (223). Similarly, pomalidomide has shown to repolarize macrophages from M2 to M1 and increase their phagocytic activity in mouse models of PCNSL, which is probably mediated by the potentiated STAT1 signaling while inhibited STAT6 signaling (35).

Therapeutically, macrophages possess immense potential of eliciting antibody-dependent cellular phagocytosis (ADCP) to destroy tumor cells (224). Of note, ADCP was demonstrated as one of the driving cytotoxic mechanism for anti-CD20 and anti-CD38 therapeutic antibodies against B-cell neoplasms (224, 262, 263). Thus, harnessing and enhancing macrophage-mediated ADCP through repolarization of M1/M2 macrophages is poised to become a novel and effective strategy for immunotherapy. Lenalidomide was shown to improved MOR202 (an anti-CD38 mAb)-mediated tumoricidal activity of MAMs against primary MM cells by restoring the defective vitamin D pathway in these MAMs with reduced CYP27B1 level (225). In addition, lenalidomide and pomalidomide mediated a substantial CD38 upregulation on MM cell lines, which also contributes to a synergistic enhancement of cytotoxic activity by combining MOR202 with IMiDs (213). Despite the enhanced ADCP of anti-CD20 mAbs by IMiDs has not been fully studied, it deserves further investigation for clinical application especially considering that obinutuzumab, the third-generation type II humanized anti-CD20 mAb (264), has shown to induce stronger ADCP as compared to rituximab, which may be due to the increased activation of FcγRI (CD64) expressed on primary macrophages (226).

#### 3.2.4 Effects on DCs

As the most powerful antigen presenting cells (APCs), DCs are key messengers and link between the innate and adaptive immune systems by capturing and presenting tumor antigens for T-cell recognition (265, 266). Evidence of immunomodulatory activity of IMiDs on DCs was first revealed in mouse, showing that lenalidomide and pomalidomide upregulated MHC class I molecules and CD86 on DCs derived from bone marrow, promoted antigen uptake antigen and presentation of DCs for naive CD8^+^ T cells (229). Pomalidomide can also increase the expression of MHC class II molecules on DCs, resulting in increasing CD4^+^ T cell priming (229). Recently, Phan et al. showed that IMiDs have the potential to shift the DC-mediated response from Th1 to Th2 humoral immunity in human. IMiDs potentially enhanced DC-mediated allergic Th2 responses (CCL17 secretion and memory Th2 response) through upregulated STAT6 and IRF4 (267). Interestingly, high CCL17 levels in serum at the onset of rash as a side effect correlate with clinical outcome of lenalidomide treatment, which suggests that DCs immunostimulation inextricably linked side effect and activity of IMiDs (267). These findings also provide evidence for the additional use of IMiDs in dendritic cell–based anti-tumor vaccines (230, 231).

#### 3.2.5 Effects on stromal cells and angiogenesis

In pathological conditions, malignant B cells rely on interactions with nonmalignant stromal cells within bone marrow and secondary lymphoid organs for their survival and proliferation (237). In MM, cytokines derived from bone marrow-derived mesenchymal stromal cells (BMSCs), an integral part of the non-hematopoietic BM microenvironment, are considered important drivers of myeloma pathobiology (268). Treatment with IMiDs significantly abrogates the interaction between MM cells and BMSCs by decreasing the production of IL-6 by stromal cells and downregulating adhesion molecules including LFA-1/ICAM-1 and VLA-4/VCAM-1 (269). In addition, lenalidomide potentially inhibits the pro-survival activity of BMSCs in MCL by inhibiting IL-6-mediated STAT-3 signaling (270). Lenalidomide may also target CXCL12/CXCR4 axis by inhibiting production of CXCL12 by MSCs in NHL (271). To date, the exact impacts of IMiDs on other nonimmune components of TME in B-cell neoplasms such as cancer-associated fibroblasts (CAFs), extracellular matrix (ECM) and pericytes, are still unknown.

Angiogenesis is a constant hallmark from initiation to progression for both MM and BCLs (272, 273). The antiangiogenic activity of IMiDs have been well characterized in MM, which was initially thought as the major MOA of thalidomide analogs against myeloma progression (274). Thalidomide impairs angiogenesis *via* suppression of vascular endothelial growth factor (VEGF) signaling (275). Similarly, lenalidomide exerts anti-angiogenic activity by downregulating basic fibroblast growth factor (bFGF) and VEGF due at least in part to inhibition of Akt phosphorylation (276). In CLL, lenalidomide was shown to inhibit CLL-mediated pro-angiogenic effect *in vitro* and modulates angiogenesis-related factors in patients with R/R CLL (277). Moreover, lenalidomide also exhibits inhibitory effects on VEGF-mediated angiogenesis and lymphangiogenesis in mouse models of B-cell lymphoma (64).

## 4 IMiDs in the era of immunotherapy

### 4.1 Antibody-based therapies

Due to extensive capacity of antibodies for targeting tumor-specific antigens, antibody-based therapies have become the most frequently used immunotherapeutic method for cancer treatment. The potent anti-tumor activity of rituximab in patients with various lymphoid malignancies has led to its widespread use in most indolent and aggressive CD20^+^ BCLs (278). As shown in preclinical studies exhibiting synergistic anti-tumor activity, the chemotherapy-free combination of rituximab plus lenalidomide (R^2^ regimen) proved to be effective in previously untreated indolent lymphoma (FL and MZL) and induced high molecular response (25, 279, 280). Similarly, obinutuzumab plus lenalidomide (GALEN regimen) has also been demonstrated as an active immunomodulatory combination with a manageable safety profile in both front-line and R/R FL (133, 281). Although the MOA of obinutuzumab favors it as a more effective anti-CD20 mAb (264), it remains uncertain whether rituximab or obinutuzumab is the better one when combined with lenalidomide in indolent lymphoma. In CLL, the combination of lenalidomide and ofatumumab was well-tolerated and induced durable responses in the majority of R/R patients with 71% ORR and a long progression-free survival (PFS) of 16 months (282). The ability to augment ADCC and ADCP suggests that lenalidomide should also cooperate with other therapeutic antibodies beyond anti-CD20 mAbs. Daratumumab (an anti-CD38 mAb) is approved as monotherapy or in combination with standard regimens for treatment of newly diagnosed (ND) or R/R MM (214). In RRMM, daratumumab in combination with dexamethasone and lenalidomide led to a significant PFS benefit over dexamethasone and lenalidomide alone (215, 283). The phase 3 MAIA study further demonstrated that daratumumab plus dexamethasone and lenalidomide increased OS and PFS of NDMM patients ineligible for transplantation (120). In addition, the anti-CD19 mAb MOR-28 (Tafasitamab) plus lenalidomide has shown outstanding clinical benefits with durable response rates in a phase 2 trial for R/R DLBCL (28).

Bi-specific T-cell engagers (BiTEs) are a new category of artificial bispecific antibodies (BsAbs) engineered to recognize specific tumor-associated antigen and CD3 at the same time (284, 285). Given the promising clinical efficacy of BiTEs in R/R BCLs (286), the combinations of lenalidomide with BsAbs such as Blinatumomab (a CD19/CD3 BiTE)and Mosunetuzumab (a CD20/CD3 BiTE) are currently being investigated in early-phase 1 clinical trials (209–211).

### 4.2 ICIs

The use of ICIs targeting PD-1 signaling pathway has ushered in a paradigm shift in cancer due to success in various high-risk solid tumors (287). However, the activity of ICIs in hematologic malignancies is currently restricted to certain subtypes of lymphoma, such as Hodgkin lymphoma (HL) and primary mediastinal B-cell lymphoma (PMBCL) (288). The severe T-cell tolerance and exhaustion within the TME is considered as the major contributor to disappointing clinical results for anti-PD-1 monotherapy in NHLs and CLL (289, 290). A recent study by Geng et al. showed that lenalidomide bypasses the requirement of CD28 for tumor-infiltrating CD8^+^ T-cell activation and antitumor activity of PD-1 blockade, which suggests that lenalidomide combination is beneficial to overcome PD-1 resistant tumors infiltrated with CD28^-^ exhausted T cells (206). In addition, another preclinical study demonstrated avadomide combination enhanced anti-CLL activity of anti-PD-1/PD-L1 therapy (245). Mechanistically, avadomide stimulated T-cell activation, motility, cytokine production, IS formation, and IFN‐γ‐inducible expression of PD‐L1, thus reshaping a non-T cell-inflamed into a T cell-inflamed TME (245). Moreover, single blockade of PD-1 or dual blockade using anti-PD-1/PD-L1 antibodies plus lenalidomide blocked the cross-talk between myeloma cells and BMSC, thus inducing an anti-myeloma immune response to inhibit cell growth (291). Despite some early-phase 1/2 trials of pembrolizumab (an anti-PD-1 mAb) plus IMiDs and dexamethasone reported a ~50% ORR in patients with RRMM (292–294), however, phase 3 trials (KEYNOTE-183 and KEYNOTE-185) evaluating the combination of pembrolizumab with dexamethasone and an IMiD in RRMM (with pomalidomide) and NDMM (with lenalidomide) was eventually discontinued due to higher risk of death (295, 296). Further studies are needed to determine the mechanism underlying the unexpected toxicity, which will contribute to realize the therapeutic potential of ICIs and IMiDs combination in the clinic.

### 4.3 CAR-T cell therapy

CAR-T cell therapies have been approved for treatment of R/R B-ALL and aggressive B-NHLs. There are intensive bench-to-bedside studies underway to further improve the efficacy of CAR-T cells, focusing on recently described resistance mechanisms, such as T-cell exhaustion, immunosuppressive TME, defective IS, downregulation of target antigens, among others (297, 298). A strong rationale supports the combination of IMiDs and CAR-T therapy according to the enhanced activity of effector T cells and other cellular components in the TME re-educated by IMiDs. *In vivo* models have demonstrated that lenalidomide significantly enhances anti-lymphoma functions of CD19 and CD20 CAR-T cells, with decreased tumor burden and increased intratumoral CD8^+^ T cells (207). Another study showed that lenalidomide improved the efficacy of CS1-directed CAR-T cells against MM by enhancing expansion, cytotoxicity, memory maintenance, Th1 cytokine production, and IS formation of CAR-T cells (208). In addition, lenalidomide has shown to maintain the *in vitro* activity of CD23 CAR-T cells, preserve functional CAR T-CLL cell immune synapses, and improve the therapeutic efficacy of CD23 CAR-T cells *in vivo* (73). Despite the evidence of synergistic efficacy, it should be noted that the specific toxicities associated with CAR-T cells plus IMiDs, such as severe cytopenias and cytokine release syndrome (299, 300), will need to be carefully examined. Current ongoing trials have included the combing IMiDs with CD19 or B cell maturation antigen (BCMA) CAR-T cell therapy in DLBCL and MM (301–304).

### 4.4 Conventional chemotherapy

Despite advances in treatment, conventional chemotherapy is still the mainstay to induce a fast clinical remission of most hematologic cancers in the age of targeted and immune therapies. The introduce of IMiDs to chemotherapy regimen for decades has dramatically increased CR ratio and improved prognosis of NDMM (121, 274). Currently, induction treatments for MM have traditionally relied on a backbone of a combinations of IMiDs (thalidomide, lenalidomide and pomalidomide), proteasome inhibitors, alkylators (or anthracyclines), and/or steroids (274). In this scenario, IMiDs are believed to improve the immune environment beyond direct anti-tumor activity, which ensures persistent minimal residual disease (MRD) negativity through enhanced immunological surveillance against myeloma cells (305). In addition, the recently approved anti-CD38 antibodies have also shown to reshape the MM immune environment *via* activation of T and NK cells and suppression of Tregs (305). These combined immunogenic chemotherapies are paving a promising way to “cure MM”. Similarly, adding lenalidomide to R-CHOP (rituximab plus cyclophosphamide, doxorubicin, vincristine, and prednisone) (R^2^-CHOP regimen) has recently shown improved outcomes in ABC-type DLBCL (306). As such, a deeper understating of immune dysfunction in B-cell malignancies has already led to the development of a more effective and less toxic immunotherapy-chemotherapy combinations to be given to cancer patients.

## 5 Conclusions and perspectives

Compelling evidence over last decades has shown the potent immunomodulatory effects of IMiDs on diverse cellular components (T cells, NK cells, TAMs, DCs, etc.) that reside within TMEs of B-cell neoplasms, which repurposes these agents to play a role in the era of immunotherapy (**Table 2**). The promising outcomes of chemotherapy-free regimen combining IMiDs with mAbs (e.g. rituximab or obinutuzumab) in treatment of both indolent and aggressive NHL types exemplify a shift of paradigm from the standard chemotherapy to a safer and more effective IMiD-intensified immunotherapy. Based on these findings in hematologic cancers, a number of studies have explored the potential applications of IMiDs in solid tumors. For instance, CC-885, a novel CRBN modulator, has shown to induce CRBN- and p97-dependent PLK1 degradation and synergizes with volasertib (PLK1 inhibitor) to suppress lung cancer (307). Moreover, pomalidomide can generate an immune-responsive and anti-tumorigenic environment and provide an ideal combination treatment with chemotherapeutic drugs or other immunotherapies in pancreatic cancer (308). Other studies also reported activities of lenalidomide in breast cancer (309), prostate cancer (310) and colon adenocarcinoma (206). Although IMiDs by themselves exhibit very limited anti-tumor activity against solid tumors in the clinic (311), their broad immunobiological properties revert the immune regulatory milieu of TME and create opportunities for other therapeutics to achieve better responses (206).

Of note, despite a series of preclinical studies have shed novel light on the synergistic effects and MOA, the clinical safety and efficacy of the combination of IMiDs with other novel immunotherapies such as BiTEs, ICIs and CAR-T cell therapy are not yet fully determined. In addition, since all MM patients inevitably develops resistance to IMiDs over time, it is a significant limitation and challenge for clinicians to make decisions about RRMM treatment. From a molecular point of view, IMiD resistance involves downregulation of CRBN expression, IKZF1/3 and CRBN mutations, deregulation of IRF4 expression, abnormal epigenetic mechanisms (CBP/EP300, BRD4 and HDAC) and aberrant signaling pathways (Wnt, STAT3 and MAPK/ERK) (312, 313). Fortunately, recent studies have discovered that some potential novel agents and PROTACs, which target the resistance mechanisms, can increase the sensitivity of MM cells to IMiDs or synergistically enhance the anti-myeloma activity of IMiDs (313). Further studies to verify the safety and efficacy of these strategies in clinic are urgently needed to pave the way for the treatment of R/R settings. Moreover, although the E3 ubiquitin ligase CRBN is now considered as the major target that likely underlies the effects of IMiDs in tumor cells as well as immunomodulation, there are a range of key issues be addressed including: 1) the functions of CRBN in the absence of IMiDs and its physiological significance is still unknown; 2) the common and distinct neosubstrates of CRBN in tumor cells and immune cells are not fully identified; 3) the CRBN-independent mechanisms underlying the anti-tumor and immunomodulatory activities of IMiDs are reported and merit in-depth investigation. Further elucidation of these issues will contribute to optimize IMiDs-based immunotherapeutic combinations and overcome intractable drug resistance.

## Author contributions

KZ conceived and designed the review. HG drafted and revised the manuscript. JY and HW helped with the literature collection. XL and YL proofread the manuscript and provided suggestions. All authors contributed to the article and approved the submitted version.

## Funding

This study was supported by the National Natural Science Foundation of China (No. 81470336 to KZ).

## Conflict of interest

The authors declare that the research was conducted in the absence of any commercial or financial relationships that could be construed as a potential conflict of interest.

## Publisher’s note

All claims expressed in this article are solely those of the authors and do not necessarily represent those of their affiliated organizations, or those of the publisher, the editors and the reviewers. Any product that may be evaluated in this article, or claim that may be made by its manufacturer, is not guaranteed or endorsed by the publisher.

## Abbreviations

AML, Acute myeloid leukemia; ADCC, Antibody-dependent cell-mediated cytotoxicity; ADCP, Antibody-dependent cellular phagocytosis; APC, Antigen presenting cell; ASCT, Autologous stem cell transplantation; BCL, B-cell lymphoma; BsAb, Bispecific antibody; BiTE, Bi-specific T-cell engager; BMSC, Bone marrow-derived mesenchymal stromal cell; CK1α, Casein kinase 1 alpha; CRBN, Cereblon; CAR, Chimeric antigen receptor; CLL, Chronic lymphocytic leukemia; CR, Complete response; DC, Dendritic cell; DLBCL, Diffuse large B-cell lymphoma; FDC, Follicular dendritic cell; FL, Follicular lymphoma; FDA, Food and Drug Administration; ICI, Immune checkpoint inhibitor; IS, Immune synapse; IMiD, Immunomodulatory drug; IRF4, Interferon regulatory factor 4; MCL, Mantle cell lymphoma; MOA, Mechanism of action; mAb, Monoclonal antibody; MM, Multiple myeloma; MDS, Myelodysplastic syndrome; NK, Natural killer; ND, Newly diagnosed; NHL, Non-Hodgkin lymphoma; ORR, Overall response rate; PDX, Patient-derived xenograft; PCNSL, Primary central nervous system lymphoma; PEL, Primary effusion lymphoma; PFS, Progression-free survival; Treg, Regulatory T cell; R/R, Relapsed/refractory; SLE, Systemic lupus erythematosus; TME, Tumor microenvironment; TAA, Tumor-associated antigen; TAM, Tumor-associated macrophage; VEGF, Vascular endothelial growth factor.
